# **mhn** : a Python package for analyzing cancer progression with Mutual Hazard Networks

**DOI:** 10.1093/bioadv/vbaf283

**Published:** 2025-11-10

**Authors:** Stefan Vocht, Yanren Linda Hu, Andreas Lösch, Kevin Rupp, Tilo Wettig, Lars Grasedyck, Niko Beerenwinkel, Rainer Spang, Rudolf Schill

**Affiliations:** Department of Statistical Bioinformatics, University of Regensburg, 93053 Regensburg, Germany; Department of Statistical Bioinformatics, University of Regensburg, 93053 Regensburg, Germany; Department of Statistical Bioinformatics, University of Regensburg, 93053 Regensburg, Germany; Department of Biosystems Science and Engineering, ETH Zürich, 4056 Basel, Switzerland; Faculty of Physics, University of Regensburg, 93040 Regensburg, Germany; Institute for Geometry and Applied Mathematics, RWTH Aachen, 52056 Aachen, Germany; Department of Biosystems Science and Engineering, ETH Zürich, 4056 Basel, Switzerland; Department of Statistical Bioinformatics, University of Regensburg, 93053 Regensburg, Germany; Department of Biosystems Science and Engineering, ETH Zürich, 4056 Basel, Switzerland

## Abstract

**Summary:**

Mutual Hazard Networks (MHNs) are statistical models for analyzing (genetic) cancer progression. Many cancers develop silently and are only noticeable when they have significantly progressed, creating an observational gap until diagnosis. MHNs bridge this gap by reconstructing the underlying dynamics of disease progression. We present mhn, a Python package for dynamic cancer progression analysis using MHNs. It trains an MHN model from tumor genotypes. mhn overcomes challenges of numerical efficiency in model training by making use of *state space restriction*, allowing training MHNs with >100 mutational events, 5 times more than was possible before. The package offers (i) reconstruction of the most likely evolutionary history of tumors, (ii) sampling of artificial tumor histories, and (iii) visualization of genomic interactions and likely progression trajectories. These features substantially extend earlier implementations, providing a fast and user-friendly framework for researchers and clinicians to study cancer dynamics.

**Availability and implementation:**

mhn can be installed from PyPI using pip and is available under the MIT License on GitHub (https://github.com/spang-lab/LearnMHN). Installation instructions and package functionalities are detailed on GitHub and PyPI, with a comprehensive guide on Read the Docs (https://learnmhn.readthedocs.io/en/latest/index.html) and a Jupyter notebook on GitHub to help users explore the package.

## 1 Introduction

Cancer progression models (Beerenwinkel *et al.* 2015, Diaz-Uriarte and Johnston 2025) leverage cross-sectional event data from multiple cancer patients to infer interactions among gene alteration events and elucidate their impact on disease progression. Mutual Hazard Networks (MHNs) are progression models (Rupp *et al.* 2024, Schill *et al.* 2024) that offer a versatile framework for analyzing (genetic) cancer progression. These networks specifically accommodate mutual interactions between progression events, enhancing our understanding of how these dynamics influence disease.

In essence, MHNs use continuous-time Markov chains to model how a tumor accumulates *n* possible progression events. The model describes the progression through $2^{n}$ possible states over time, where each state represents the events that have occurred. The model assumes that every tumor starts in a healthy state with no progression events present, accumulates events irreversibly, and is eventually observed stochastically at a rate that depends on the current genotype. Each event has a specific base rate representing its spontaneous accumulation, independent of other events. Moreover, the presence of one event can modify the rates of subsequent events by some factor, following the mutual hazard assumption. Finally, detection of the tumor, and thus observation of its genome, is modeled as a separate event whose rate is affected by the presence of the standard progression events. Importantly, this resolves a pervasive collider bias in modeling cancer progression from cross-sectional data (Schill *et al.* 2024).

The model is fully specified by base rates, interactions, and observation rates, which are learned during model training, resulting in an (n+1)×n parameter matrix; for an example see Fig. 2A. For more details we refer to Schill *et al.* (2020) and Schill *et al.* (2024).

Note that the state space of the progression process grows exponentially with the number of included events. Therefore, computational efficiency becomes a significant challenge.

Here, we introduce mhn, a Python package for the efficient analysis of cancer progression using MHNs.

**Key features:** 

**Figure 1.**
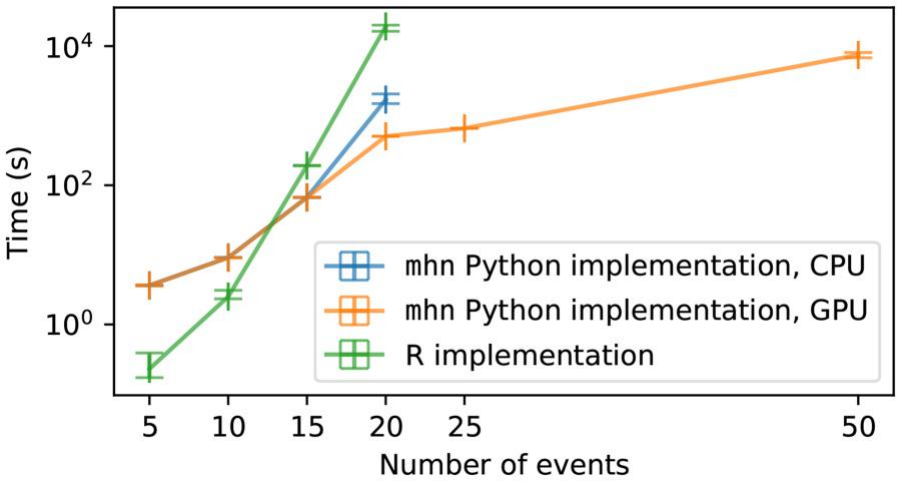
Comparison of run times for mhn and the existing R implementation. Training was performed on a dataset with 3661 samples and repeated 10 times for each configuration. mhn executes computations pertaining samples with < 13 active events on the CPU by default. Therefore, computation times for datasets with ≤15 samples are similar for the CPU and the GPU case. Especially for bigger datasets, mhn outperforms the R implementation and for 25 and 50 events, only mhn’s GPU implementation is feasible. Benchmarks were executed on a Linux computing server equipped with 512 GB RAM and 30 dedicated cores from two AMD EPYC 7453 28-Core Processors as well as an NVIDIA A100 high-performance data center GPU with 80 GB of high-bandwidth HBM2e memory.

1. **Training MHNs:** The mhn package enables users to train MHNs using their own cross-sectional datasets. Hyperparameters can be optimized through cross-validation.
2. **Visualization:** The MHN parameter matrix, i.e. base rates and interaction strengths, can be displayed in a heatmap. Moreover, the most likely trajectories in which the events of observed genotypes accumulated can be visualized in a tree.
3. **Data Generation:** From a trained MHN, one can sample artificial cancer progression data.
4. **Efficiency:** Using *state space restriction* and CUDA implementations, the computational load is managed such that, in practice, the analysis of larger datasets with over 100 events is possible as long as there is no individual sample with more than about 25 active events. The technical limit is 32 active events per observation. Note that this is not an approximation but a mathematical trick to compute the exact likelihood and gradient more efficiently. For implementation details see Appendix 1. This makes mhn outperform the existing implementation (Schill *et al.* 2020) and makes the analysis of significantly bigger datasets than before possible; see Fig. 1.

Except for the plain (not cross-validated) training, all of these features are newly introduced with the mhn package.

## 2 Cancer progression analysis with mhn

In the following, we demonstrate mhn on a dataset of 12 progression events in 3662 primary lung adenocarcinomas (LUADs) derived from targeted clinical sequencing at the Memorial Sloan Kettering Cancer Center (AACR-GENIE-Consortium 2023). Specifically, these binary events represent single-nucleotide variants (SNVs) in 12 cancer driver genes (collapsed per gene), but other alteration types like copy number changes are also possible. For preprocessing details and a more comprehensive analysis of the same dataset, see Schill *et al.* (2024).

### 2.1 Training an MHN

*Loading training data:* MHNs are trained on cross-sectional data. Specifically, mhn expects a binary matrix as input where rows correspond to observations (e.g. cancer patients/samples) and columns correspond to events (e.g. specific driver gene mutations). Entries are 1 if the respective event is present in the respective observation and 0 otherwise. Such matrices can be handed over to mhn in many formats, including CSV files, NumPy (Harris *et al.* 2020) arrays and Pandas (McKinney 2010) DataFrames, enabling seamless integration with different preprocessing workflows.

*Selecting a regularization penalty:* Users can choose from several regularization penalties. These are:

- **L1 penalty:** Encourages sparsity in the parameter matrix by driving smaller parameter values to zero. This promotes simpler, more interpretable models where only the most critical event interactions remain active.
- **L2 penalty:** Penalizes large parameter values, encouraging small, non-zero coefficients. Unlike the L1 penalty, it is suitable when most event interactions are expected to have small effects.
- **Symmetric penalty:** Promotes symmetry in the parameter matrix so that both interactions between an event pair are driven towards similar values unless the data clearly suggests dissimilarity. This is useful if one in general expects that effects between event pairs are reciprocal and roughly equal in strength. This is further discussed in Schill *et al.* (2024).
- **Custom penalties:** Advanced users may define their own custom penalty function. This flexibility allows for specialized constraints that may be tailored to specific datasets or biological assumptions. Custom penalties can be provided as callable Python functions, enabling full control over the regularization behavior.

Furthermore, mhn implements cross-validation to determine the best penalty strength. Note that, in the generally discouraged unregularized case, machine imprecisions can lead to slightly different results between training with the CPU and the GPU.

*Backwards compatibility:* Additional to training an MHN as described above (Schill *et al.* 2024), one can train previous versions of MHN (Schill *et al.* 2020) that do not explicitly model the observation event, allowing users to reproduce old results and compare them to newer methodologies.

### 2.2 Interpreting an MHN

**Figure 2.**
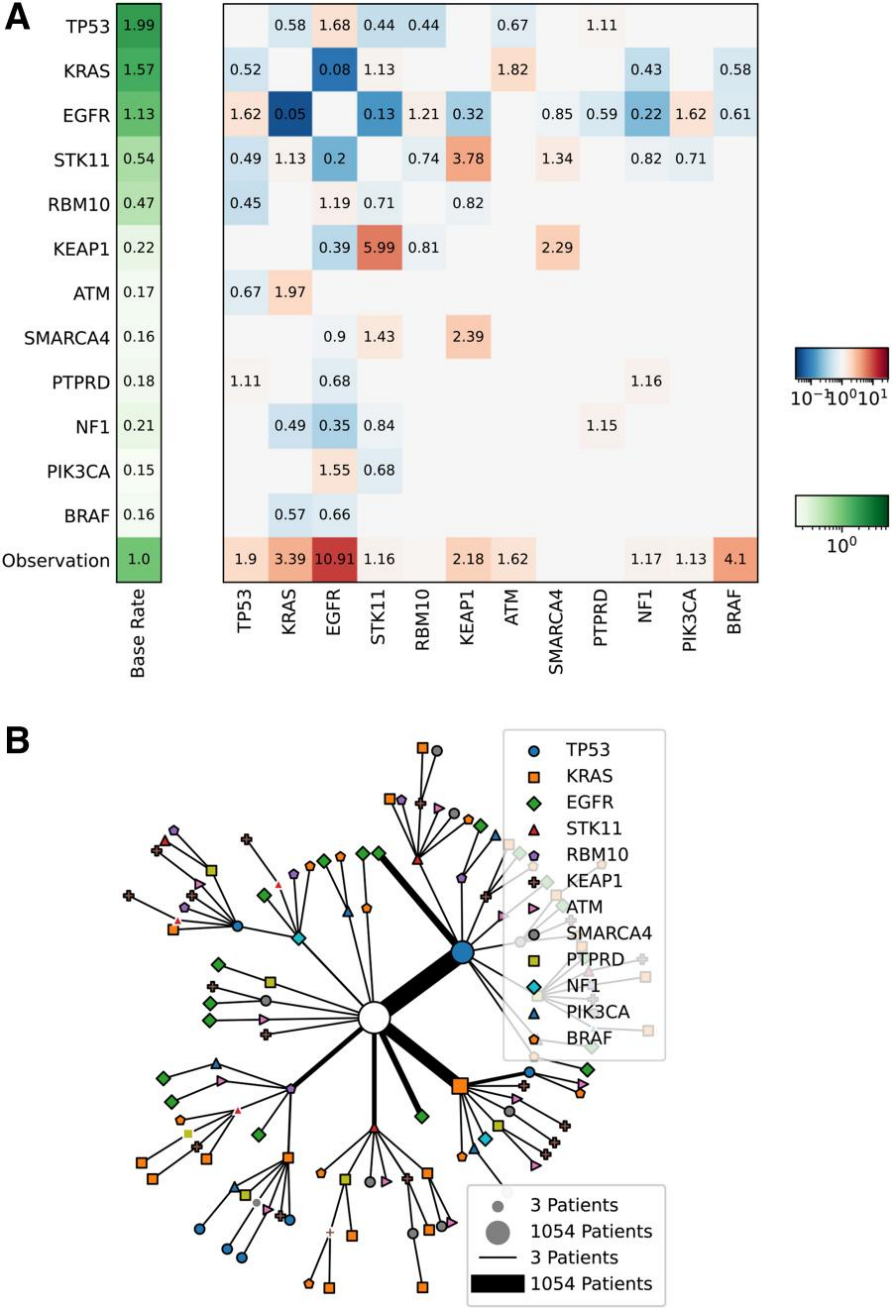
(A) Heatmap visualizing an MHN that was trained on 12 driver mutations of 3662 primary lung adenocarcinomas. Numbers in the cells correspond to the base rates (left column) and rate multipliers (matrix). The bottom row indicates the influence on the observation rate each event has. In this example, EGFR increases the observation rate by a factor of 10.91. Furthermore, the model suggests a synergy between EGFR and TP53, in which the presence of one event promotes the other’s rate. At the same time, we see a suppressive relationship between EGFR and KRAS. (B) Most likely chronological sequences of events for the most common cancer genotypes as inferred by the MHN. Each path from the root of the tree (white circle) to a leaf illustrates a possible cancer progression within the dataset. The symbols along each path denote events whose most probable chronological order was derived from the trained model. Each cancer state corresponds to a terminal node in the tree or an internal node with a black outline. To reduce visual clutter, only cancer states shared by at least three patients are depicted. The size of the edges and symbols along a path scale with the total number of patients with that cancer state. Most cancer event sequences start with either TP53 or KRAS and many end with EGFR.

An MHN is represented by a matrix that holds the base rates of events and their interactions, as well as observation rates. Diagonal entries represent the base rates of individual events. Off-diagonal entries indicate how the occurrence of one event (column) influences the rate of another event (row). The mhn package includes a method to visualize this matrix, where the base rates are shown separately (Fig. 2A).

### 2.3 Sampling artificial cancer histories

mhn can sample artificial cancer progression data from a given MHN. It also allows for keeping track of the ordering in which events accumulate, giving users the possibility to answer questions about the order of event accumulation.

### 2.4 Reconstructing cancer histories

Given a set of genotypes, the mhn package can reconstruct the most probable sequence of mutational events leading to the observed data for a given MHN. This feature is crucial for understanding the temporal dynamics of cancer progression. Additionally, the package allows users to visualize these chronological sequences, as illustrated in Fig. 2B.

## 3 Availability and documentation

mhn is available under the MIT License and can be installed from PyPI using pip. Comprehensive documentation, including installation instructions and usage examples, is provided on GitHub (https://github.com/spang-lab/LearnMHN) and Read the Docs (https://learnmhn.readthedocs.io/en/latest/index.html). A Jupyter notebook is available on GitHub to guide new users through the package’s functionalities.

## 4 Conclusion

The mhn package offers a versatile tool for the analysis of cancer progression using Mutual Hazard Networks. By addressing computational challenges, it enables the efficient analysis of large datasets. It provides researchers and clinicians with deep and quantitative insights into the complex mechanisms of cancer progression.

## Figures and Tables

**Figure 3.**
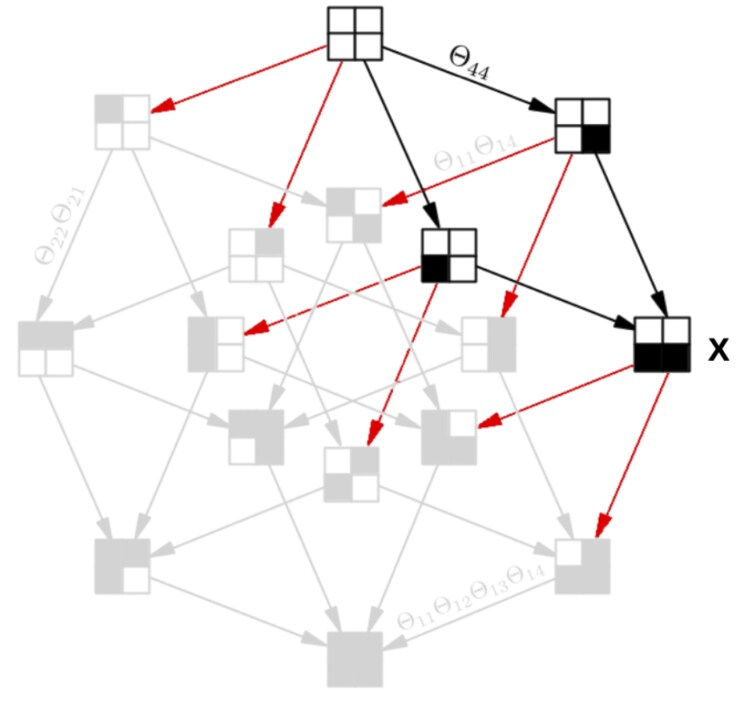
Visualization of *state space restriction* for a four-event MHN: In this case we have x=(0,0,1,1). Only states whose active events are also active in x are considered. The red arrows indicate the transitions from states in $S_{x}$ to states outside of $S_{x}$ that have to be accounted for to make the restricted state space compatible with the full MHN state space. Figure taken from Schill, 2022.

## Data Availability

The data underlying this article are available at https://github.com/spang-lab/LearnMHN/.

## Appendix 1: Efficient training with State Space Restriction

Here, we will give some details on the mathematics behind and implementation of the mhn package as well as its performance. We will do so on the basis of a previous version of an MHN, which we will denote by cMHN, for classical MHN. This can then easily be generalized to the current version of an MHN.

A cMHN is a matrix $(\Theta_{ij})\in R^{n\times n}$ that parametrizes the transition rate matrix $Q\in R^{2^{n}\times 2^{n}}$ of a continuous-time Markov process over all $2^{n}$ combinations of *n* predefined binary events. It does so via a sum of tensor products:

(A.1)
$$Q=\sum_{i=1}^{n}\overset{i-1}{\underset{j=1}{⊗}}\left(\begin{array}{cc} 1 & 0\\ 0 & \Theta_{ij} \end{array}\right)⊗\left(\begin{array}{cc} -\Theta_{ii} & 0\\ \Theta_{ii} & 0 \end{array}\right)⊗\overset{n}{\underset{j=i+1}{⊗}}\left(\begin{array}{cc} 1 & 0\\ 0 & \Theta_{ij} \end{array}\right).$$

In a cMHN, the observation time is treated as an exponentially distributed random variable with mean 1. The probability distribution, marginalized over the observation time, of all states of the Markov process $p_{\Theta }$ can be calculated as

(A.2)
$$p_{\Theta }={\left(I-Q\right)}^{-1}p_{\emptyset },$$

where we have $p_{\emptyset }:=(1,0,\ldots ,0)$ as the initial distribution concentrated in the initial state without any active events. To train a cMHN on a given dataset D, we typically maximize the marginal log-likelihood

(A.3)
$$\ell_{D}=p_{D}^{T}\;\text{log}\;p_{\Theta }=p_{D}^{T}\;\text{log}\;\left({\left(I-Q\right)}^{-1}p_{\emptyset }\right).$$

Here, $p_{D}$ is the empirical probability distribution given by the dataset D.

As (I−Q) is a lower triangular matrix, we can use forward-substitution to compute ${\left(I-Q\right)}^{-1}p_{\emptyset }$ more efficiently. This and exploiting the way how *Q* is constructed from $(\Theta_{ij})\in R^{n\times n}$ yields a runtime of $O(n^{2}2^{n})$ for computing the marginal log-likelihood. This approach is significantly better than the naive approach of computing the log-likelihood on the actual transition rate matrix *Q* (runtime of $O(2^{2n})$), but still makes training MHNs for more than about 25 events practically infeasible.

To solve this issue, we introduce *state space restriction* and only focus on events that are actually present in a state in D. This idea already appears in Gotovos *et al.* (2021); Chen (2023), but here we also provide tensor representations of the restricted transition rate matrix which allows for efficient computations.

Let x be a state that is contained in dataset D and $S_{x}$ be the set of all states whose active events are also active in x. Let $Q_{x}$ be the principal submatrix of *Q* over all states in $S_{x}$. This means that $Q_{x}$ contains all transitions between the states in $S_{x}$, but, crucially, also accounts for all transitions that lead from states inside $S_{x}$ to states that lie outside of $S_{x}$ (Fig. 3). Mathematically, $Q_{x}$ can be expressed as

(A.4)
$$Q_{x}=\sum_{i}\underset{j}{⊗}{\left(Q_{x}\right)}_{i}^{\left(j\right)}$$

with

(A.5)
$${\left(Q_{x}\right)}_{i}^{\left(j\right)}=\{\begin{array}{cc} \left(\begin{array}{cc} 1 & 0\\ 0 & \Theta_{ij} \end{array}\right) & \text{if}\;i\neq j\;\text{and}\;x_{j}=1,\\ \left(\begin{array}{c} 1 \end{array}\right) & \text{if}\;i\neq j\;\text{and}\;x_{j}=0,\\ \left(\begin{array}{cc} -\Theta_{ii} & 0\\ \;\Theta_{ii} & 0 \end{array}\right) & \text{if}\;i=j\;\text{and}\;x_{j}=1,\\ \left(\begin{array}{c} -\Theta_{ii} \end{array}\right) & \text{if}\;i=j\;\text{and}\;x_{j}=0. \end{array}$$

Notice that, for *m* being the number of active events in x, we have $Q_{x}\in R^{2^{m}\times 2^{m}}$. The marginal log-likelihood for x can then be computed as

(A.6)
$$\ell_{x}={\left(0,\ldots ,0,1\right)}^{T}\;\text{log}\;\left({\left(I-Q_{x}\right)}^{-1}p_{\emptyset }\right),$$

where we abuse notation to write $p_{\emptyset }$ for the restriction of $p_{\emptyset }$ to $R^{2^{m}}$. Computing $\ell_{x}$ has a runtime of $O(nm2^{m})$. The log-likelihood for the whole dataset is then simply a normalized sum over the log-likelihoods of all states present in D:

(A.7)
$$\ell_{D}=\frac{1}{\left|D\right|}\sum_{x\in D}\ell_{x}.$$

Let $m_{\text{max}}$ be the maximum number of active events that is present in a single sample of D. Then the runtime of computing $\ell_{D}$ is in $O(nm_{\text{max}}2^{m_{\text{max}}}|D|)$. This means that, using *state space restriction*, training much larger MHNs becomes feasible as long as there is no state in dataset D with more than about 25 active events.
